# Role of Strain Imaging in Right Heart Disease: A Comprehensive Review

**DOI:** 10.14740/jocmr1842w

**Published:** 2014-07-28

**Authors:** Arun Kannan, Chithra Poongkunran, Mahendran Jayaraj, Rajesh Janardhanan

**Affiliations:** a Section of Inpatient Medicine, 1501 N Campbell Ave, PO Box 245212, Tucson, AZ 85724, USA; b Non-Invasive Cardiac Imaging, Sarver Heart Center, University of Arizona, Box 245037, 1501 N Campbell Ave, Tucson, AZ 85724, USA

**Keywords:** Speckle tracking, Strain imaging, Right ventricle, Pulmonary hypertension, ARVD

## Abstract

Advances in the imaging techniques of the heart have fueled the interest in understanding of right heart pathology. Recently, speckle tracking echocardiography has shown to aid in understanding various right heart diseases and better management. Its role is well established in diagnosing right heart failure, pulmonary artery hypertension, arrhythmogenic right ventricular dysplasia and congenital heart disease. We review the basic mechanics of speckle tracking and analyze its role in various right heart conditions.

## Introduction

For the past three decades, the left ventricular anatomy and function has been extensively researched and studied. The right ventricle (RV) has been ignored probably due to the technical difficulties in imaging as well as the poor understanding of its function and hemodynamics. Recent advances in medicine led to the better understanding of the role of RV in various medical conditions including congenital heart disease, pulmonary hypertension, arrhythmogenic right ventricular dysplasia and right ventricular myocardial infarction. This provided the impetus to focus on the management of various right heart pathologies to prevent unfavorable outcomes, like right heart failure, hemodynamically significant ventricular arrhythmias and pulmonary hypertension. RV is involved in several cardiac conditions and evaluation of the RV anatomy and function is an essential component of clinical management [1].

RV is being recognized as a separate entity with unique anatomy, physiology and function compared to the left ventricle. Multiple imaging modalities are used in imaging the RV to understand its anatomy and function. One of the most common imaging modality is 2D transthoracic echocardiogram, which is readily available. But its utility is limited by technical difficulty in imaging the RV secondary to various factors including patient’s body habitus sonographer’s skills and expertise and the dependency on angle. In this article, the role of speckle tracking in various right ventricular pathologies is reviewed.

## Speckle Tracking

Strain imaging is a novel method that has been developed to quantify regional myocardial function. Myocardial strain imaging was initially obtained by tissue Doppler imaging (TDI) [2]. More recently, it is being obtained with myocardial speckle tracking using 2D echocardiography. The term strain, also known as regional deformation, describes lengthening and shortening of individual myocardial fibers. The strain rate applies to the magnitude of change of length compared to the original length of muscle fiber. The deformation could happen in different dimensions: longitudinal, radial, circumferential and rotational. The regional myocardial function is traditionally assessed by wall motion analysis, which in itself is dependent on velocity and displacement [3].

The systolic tissue velocities at any given point are described as the sum of the longitudinal shortening (deformation) of the entire myocardial wall from that point to the apex that is the sum of the regional strain rates. In normal or symmetrically diseased myocardium, the strain rate and the tissue velocity obtained by TDI are comparable. In regionally diseased myocardium such as in myocardial infarction and focal scarring, akinesia in one segment will lead to reduced velocities in normally contracting contiguous or other distinct segments due to tethering [3]. Thus, even completely akinetic segments, without deformation, can show motion.

Myocardial deformation, assessed by strain and strain rate, is more useful than wall motion analysis (velocity and displacement) for detection of regional myocardial abnormalities. Nevertheless, TDI can be used to obtain strain and strain rate.

There are multiple disadvantages to TDI imaging. One of the primary limitations of TDI derived strain imaging is angle dependency [4]. Furthermore, they are derived from one-dimensional velocity measurements whereas myocardium deforms simultaneously in three dimensions. The images have limited spatial resolution in order to get imaging at high temporal resolution [5].

Speckles are natural acoustic markers that occur naturally in the ultrasound images. They are equally distributed throughout the myocardium. Their size is about 20 - 40 pixels [6]. The geometric movement of each of these speckles represents local tissue movement. There are multiple softwares that allow spatial and temporal image processing with recognition and selection of such elements on ultrasound images. Strain and strain rate can be calculated by tracking these speckles. The advantage of speckle tracking is that it is angle independent by virtue of its tracking along the myocardial wall rather than the ultrasound waves. The images are obtained at high frame rate and only one cardiac cycle is required [5-7].

## Role of Strain Imaging in Cardiac Load

Acute increase in preload increases cardiac contractility and should theoretically cause deformation of the right ventricular myocardium. Missant et al investigated the relation between RV contractility and regional deformation [8]. They compared the strain rate and isovolumic strain acceleration with regard to sensitivity for loading conditions in the RV and noted its role in predicting RV overload scenarios. This overload dependency of deformation is a significant finding in the RV as the afterload is minimal in resting conditions and might increase as much as four-fold in various pulmonary pathologies [9].

## Role of Speckle Tracking in Various RV Pathologies

### RV function

Transthoracic echocardiography (TTE) provides a standard evaluation of RV function in most cases. There are certain limitations with TTE such as the reproducibility of different measurements and the lack of normal references based on age, BMI and gender. Also, there is poor correlation of the 2D data with 3D echocardiographic data [10, 11]. Due to the limitations described above, echocardiographic evaluation of RV function remains challenging. The parameters such as RV and right atrial area, RV free wall thickness, longitudinal and free wall shortening help to assess RV function [12]. Recent guidelines recommend using the following echocardiographic parameters as the surrogate markers of RV function: tricuspid annular plane systolic excursion, fractional area change, or RV index of myocardial performance [13, 14]. Speckle tracking circumvents some of the limitations inherent to tissue Doppler echocardiography; longitudinal strain (LS) and strain rate measurements are independent of global cardiac motion, thus allowing quantification of regional myocardial deformation in various RV segments [15]. The deformation is decreased in patients with pulmonary hypertension, systemic RV and repaired tetralogy of Fallot (TOF) [16-18].

The role of 3D speckle tracking imaging in assessing the RV function has been studied by Atsumi et al, who noted that in patients with RV dysfunction, 3D STI indicated low peak systolic area change ratio in the damaged area [19].

### Role of speckle tracking in right heart failure

Multiple studies have shown that RV ejection fraction (EF) is an independent prognostic factor in patients with chronic systolic heart failure [20, 21]. The right ventricular function is a determinant in the success of LVADs in end stage chronic systolic heart failure [22]. Recently, speckle tracking has been studied in evaluating its role in right heart failure [23]. Meris et al compared 76 patients with RV dysfunction to 100 healthy volunteers and measured peak LS and time to LS in different RV segments [24]. The authors noted close correlation between RV contractility as measured by LS and TAPSE. Also, the global and regional deformation was decreased with RV dysfunction. In patients with advanced chronic heart failure referred for heart transplantation, Cameli et al have found that RV LS is a strong predictor of outcome [25].

### Role of speckle tracking in pulmonary arterial hypertension (PAH)

PAH is characterized by chronic RV pressure overload due to pulmonary vascular obstruction either by thrombus or smooth-muscle proliferation due to various medical conditions. 2D TTE is the most common initial imaging modality used in assessing the pulmonary hypertension. Speckle tracking has been recently studied in assessing the changes in the right heart due to pulmonary hypertension. Li et al compared 42 patients with pulmonary hypertension to 31 healthy controls by assessing multiple parameters thought to be predictive of RV changes [26]. This included RV global and longitudinal peak systolic strain and strain rates at basal, mid and apical segments of RV free wall and septum. The authors noted that strain imaging predicted the impaired RV global and regional systolic function in patients with PAH. In another study, Calcutteea et al compared 35 patients with pulmonary hypertension to 20 controls [27]. The authors noted reduced basal and mid-cavity strain rate, reduced time to peak systolic strain rate at multiple RV levels: basal, mid-cavity and RVOT.

In summary, speckle tracking echocardiography (STE) has a valid and reproducible role in the assessment of RV changes in pulmonary hypertension when used in conjunction with TTE. Multiple studies studied the prognostic role of speckle tracking in patients with chronic pulmonary hypertension. In a study of 49 patients with chronic thromboembolic pulmonary hypertension, patients were divided into two groups based on their ability to complete 6-minute walk test by walking less or more than 300 m. In patients who walked less than 300 m, RV basal-lateral strain and strain rate were lower compared to patients who were able to walk more than 300 m [28]. The authors concluded that RV myocardial deformation has shown to predict prognosis. Fukuda et al compared 45 patients with PAH with 22 age-matched volunteers [29]. The authors noted that RV free wall LS is an independent echocardiographic predictor of various hemodynamic RV parameters, including mean pulmonary artery pressure, pulmonary vascular resistance and correlated with EF and RV end-systolic volume (measured by CMR). Also, the improvement in RV free wall LS correlated with 6-minute walking test in medically treated patients.

### Role of speckle tracking in arrhythmogenic right ventricular dysplasia (ARVD)

ARVD is a hereditary cardiomyopathy characterized by fibro fatty replacement of the RV myocardium that leads to ventricular arrhythmias and right ventricular failure and sudden death [30]. Echocardiography remains one of the most common diagnostic modality in patients with suspected ARVD. In a typical presentation, echocardiography may reveal an enlarged RV, with global and/or regional RV dysfunction with structural alterations [31]. Multiple parameters have also been observed that may provide clues in the diagnosis of ARVD. Lindstrom et al have noted decreased amplitude of the tricuspid annular motion and decreased early diastolic peak annular (EA) velocity [32]. The role of STE in the diagnosis of ARVD was studied by Vitarelli et al who compared 19 ARVD patients with matched controls [33]. The right ventricular systolic LS was obtained in the basal, mid and apical segments of the RV. In the ARVD group, analysis of global and regional deformation showed that the strain values were reduced significantly in all three segments in the RV free wall compared to those in the controls. Also, the left ventricular parameters showed a decreased left ventricular longitudinal and global LV strain. The authors concluded that the RV strain was significantly lower in patients with ARVD and also predicted RV dysfunction better than the 3D RV EF.

### Role of speckle tracking in congenital heart disease

The role of speckle tracking in congenital heart disease is currently in the development stage. Few studies have reviewed its ability in evaluation of complex congenital heart disease. In a recent study, Kempny quantified biventricular myocardial function in repaired TOF by comparing CMR to speckle tracking [34]. In this study comprising 28 patients and 25 controls, peak longitudinal RV strain was found to be reduced in patients compared to controls. Alghamdi et al noted that echocardiographic RV LS might be a better correlated index compared to global RV systolic function in patients with repaired TOF [35]. In another study to evaluate the feasibility of RV longitudinal peak systolic strain (LPSS) assessment in follow-up of patients with corrected TOF, 18 patients with corrected TOF underwent echocardiography twice with a time interval of 4.2 ± 1.7 years [36]. The authors noted that RV LPSS may be a sensitive marker to detect early deterioration in RV function.

In a study done in fetuses with congenital heart disease, longitudinal left ventricular and right ventricular free wall strain and the strain ratio were measured in 14 fetuses [37]. In simple shunt lesions and shunts with pulmonary stenosis or atresia, mean strain ratios were reduced, with hypoplastic left heart being the lowest and Ebstein’s anomaly being the highest. Serial measurements showed increased LV strain in aortic coarctation and aortic stenosis, and increased RV strain in pulmonary regurgitation. More studies are needed in this field to determine the role of speckle imaging in right heart disease.

## Limitations

The limitations of speckle tracking echocardiography are the dependency on strict frame rate, conceivable inaccuracies in tracing the epicardial/endocardial border in images with poor quality, and the need for sufficient experience using analysis software [38]. Also, the software to analyze the complex RV geometry properly is not widely available.

## Future Directions

STE is a promising modality in the evaluation of global and regional cardiac function. The application of speckle tracking in cardiac conditions in which present diagnostic techniques are inadequate is being studied. Also, 3D STE may offer superior spatial resolution than 2D STE. It has great potential, but is yet to gain mainstream clinical application.

## Conclusions

STE offers a promising imaging modality in various cardiac conditions. Owing to its inherent advantages including angle independency and reproducibility, it is gaining mainstream role in the world of echocardiography. With more research being focused on the clinical application of speckle tracking, it is likely to provide an advantage in better patient selection for current and newer therapies as well as in tracking disease progression, which is important in prognosis.
